# Vision-Based Detection and Distance Estimation of Micro Unmanned Aerial Vehicles

**DOI:** 10.3390/s150923805

**Published:** 2015-09-18

**Authors:** Fatih Gökçe, Göktürk Üçoluk, Erol Şahin, Sinan Kalkan

**Affiliations:** Department of Computer Engineering, Middle East Technical University, Üniversiteler Mahallesi, Dumlupınar Bulvarı No. 1, 06800 Çankaya Ankara, Turkey; E-Mails: ucoluk@ceng.metu.edu.tr (G.Ü.); erol@ceng.metu.edu.tr (E.Ş.); skalkan@ceng.metu.edu.tr (S.K.)

**Keywords:** UAV, micro UAV, vision, detection, distance estimation, cascaded classifiers

## Abstract

Detection and distance estimation of micro unmanned aerial vehicles (mUAVs) is crucial for (i) the detection of intruder mUAVs in protected environments; (ii) sense and avoid purposes on mUAVs or on other aerial vehicles and (iii) multi-mUAV control scenarios, such as environmental monitoring, surveillance and exploration. In this article, we evaluate vision algorithms as alternatives for detection and distance estimation of mUAVs, since other sensing modalities entail certain limitations on the environment or on the distance. For this purpose, we test Haar-like features, histogram of gradients (HOG) and local binary patterns (LBP) using cascades of boosted classifiers. Cascaded boosted classifiers allow fast processing by performing detection tests at multiple stages, where only candidates passing earlier simple stages are processed at the preceding more complex stages. We also integrate a distance estimation method with our system utilizing geometric cues with support vector regressors. We evaluated each method on indoor and outdoor videos that are collected in a systematic way and also on videos having motion blur. Our experiments show that, using boosted cascaded classifiers with LBP, near real-time detection and distance estimation of mUAVs are possible in about 60 ms indoors (1032×778 resolution) and 150 ms outdoors (1280×720 resolution) per frame, with a detection rate of 0.96 F-score. However, the cascaded classifiers using Haar-like features lead to better distance estimation since they can position the bounding boxes on mUAVs more accurately. On the other hand, our time analysis yields that the cascaded classifiers using HOG train and run faster than the other algorithms.

## 1. Introduction

Advances in the development of micro unmanned aerial vehicles (mUAVs), which are UAVs less than 5 kg [1], have led to the availability of highly capable, yet inexpensive flying platforms. This has made the deployment of mUAV systems in surveillance, monitoring and delivery tasks a feasible alternative. The use of mUAVs in monitoring the state of forest fires where the mission spreads over a large region and flying over the fire is dangerous [2] or in delivering packages in urban areas [3] as a faster and less expensive solution is being explored. Moreover, the widespread interest in the public has also resulted in mUAVs, which are often referred to as drones, showing up in places, such as the White House, where conventional security measures are caught unprepared [4] or in traffic accidents or in fires where the presence of mUAVs, flown by hobbyists to observe the scene, posed a danger to police and fire-fighter helicopters and resulted in delays in their deployment [5]. In all of these cases, the need for the automatic detection and distance estimation of mUAVs, either from the ground or from a flying platform (which can be another mUAV or a helicopter) against a possibly cluttered background is apparent.

The main objective of our study is the evaluation of vision as a sensor for detection and distance estimation of mUAVs. This problem poses a number of challenges: First, mUAVs are small in size and often do not project a compact and easily segmentable image on the camera. Even in applications where the camera is facing upwards and can see the mUAV against a rather smooth and featureless sky, the detection poses great challenges. In multi-mUAV applications where each platform is required to sense its neighbors and in applications where the camera is placed on a pole or on a high building for surveillance, the camera is placed at a height that is the same or higher than the incoming mUAV, and the image of the mUAV is likely to be blended against feature-rich trees and buildings, with possibly other moving objects in the background, so the detection and distance estimation problem becomes challenging. Moreover, in multi-mUAV applications, the vibration of the platform, as well as the size, power, weight and computational constraints posed on the vision system also need to be considered.

Within this paper, we report our work towards the development of an mUAV detection and distance estimation system. Specifically, we have created a system for the automatic collection of data in a controlled indoor environment, proposed and implemented the cascaded approach with different features and evaluated the detection performance and computational load of these approaches with systematic experiments on indoor and outdoor datasets.

For the cooperative operation of mUAVs and for also sensing and avoiding purposes, relative localization in 3D space, which requires the estimation of relative bearing, elevation and distance, is critical. Relative bearing and elevation can be estimated easily by detecting an mUAV in an image. However, for distance estimation, additional computation is needed. Due to the scale estimation problem in monocular vision and the excessive variability of the possible appearances of an mUAV for the same distance, the problem is challenging. Considering the demand for the distance information, we also developed a method to estimate the relative distance of a detected mUAV by utilizing the size of the detection window. We have performed indoor experiments to evaluate the performance of this approach in terms of both distance and time-to-collision estimation.

## 2. Related Studies

In this section, we discuss the relevant studies in three parts. In the first part, general computer vision approaches related to object detection and recognition are reviewed. The second and third parts summarize the efforts in the robotics literature to detect and localize mUAVs using computer vision and other modalities, respectively.

### 2.1. Object Detection and Recognition Approaches with Computer Vision

In computer vision and pattern recognition (CVPR), object detection and recognition has been extensively studied (see [6,7] for comprehensive reviews), with applications ranging from human detection, face recognition to car detection and scene classification [8,9,10,11,12,13]. The approaches to detection and recognition can be broadly categorized into two: keypoint-based approaches, and hierarchical and cascaded approaches.

#### 2.1.1. Keypoint-Based Approaches

In keypoint-based methods, CVPR usually detects salient points, called interest points or keypoints, in the “keypoint detection” phase. In this phase, regions in the image that are likely to have important information content are identified. The keypoints should be as distinctive as possible and should be invariant, *i.e.*, detectable under various transformations. Popular examples of keypoint detectors include fast corner detection (FAST) [14,15], Harris corner detection (HARRIS) [16], maximally stable extremal region extractor (MSER) [17] and good features to track (GFTT) [18] (see [19] for a survey of local keypoint detectors).

In the next phase of keypoint-based approaches, intensity information at these keypoints is used to represent the local information in the image invariant to transformations, such as rotation, translation, scale and illumination. Examples of the keypoint descriptors include speeded-up robust features (SURF) [20], scale-invariant feature transform (SIFT) [21], binary robust independent elementary features (BRIEF) [22], oriented FAST and rotated BRIEF (ORB) [23], binary robust invariant scalable keypoints (BRISK) [24] and fast retina keypoint (FREAK) [25].

Extracted features are usually high dimensional (e.g., 128 in the case of SIFT, 64 in SURF, *etc.*), which makes it difficult to use distributions of features for object recognition or detection. In order to overcome this difficulty, the feature space is first clustered (e.g., using k-means), and the cluster labels are used instead of high-dimensional features for, e.g., deriving histograms of features for representing objects. This approach, called the bag-of-words (BOW) model, has become very popular in object recognition (see, e.g., [26,27,28]). In BOW, histograms of cluster labels are used to train a classifier, such as a naive Bayes classifier or a support vector machine (SVM) [29], to learn a model of the object.

In the testing phase of BOW, a window is slid over the image, and for each position of the window in the image, a histogram of the cluster labels of the features in that window is computed and tested with the trained classifier. However, the scale of the window imposes a severe limitation on the size of the object that can be detected or recognized. This limitation can be overcome to only a certain extent by sliding windows of different scales. However, this introduces a significant computational burden, making it unsuitable for real-time applications.

#### 2.1.2. Hierarchical and Cascaded Approaches

A better approach in CVPR is to employ hierarchical and cascaded models into recognition and detection. In such approaches, shape, texture and appearance information at different scales and complexities is processed, unlike the regular keypoint-based approaches. Processing at multiple levels has been shown to perform better than the alternative approaches (see, e.g., [30]).

In hierarchical approaches, such as the deep learning approaches [31], features of varying scale are processed at each level: in lower levels of the hierarchy, low-level visual information, such as gradients, edges, *etc.*, are computed, and with increasing levels in the hierarchy, features of the lower levels are combined, yielding corners or higher-order features that start to correspond to object parts and to objects. At the top of the hierarchy, object categories are represented hierarchically. For detecting an object in such an approach, the information needs to pass through all of the hierarchies to be able to make a decision.

An alternative approach is to keep a multi-level approach, but prune processing as early as possible if a detection does not seem likely. Such cascaded approaches, which are inspired, especially, from ensemble learning approaches [32] in machine learning, perform quickly, but coarse detection at early stages and only candidates passing earlier stages pass on to higher stages where finer details undergo computationally-expensive detailed processing. This way, these approaches benefit from speed ups by processing candidate regions that are highly likely to contain a match [33]. A prominent study, which also forms the basis of this study, is the approach by Viola and Jones [10,34], which builds cascades of classifiers at varying complexities using Haar-like features and adopting the AdaBoost learning procedure [35]. Viola and Jones [10,34] applied their method to face detection and demonstrated high detection rates at high speeds. The approach was later extended to work with local binary patterns for face recognition [36] and histogram of oriented gradients for human detection [37], which are more descriptive and faster to compute than Haar-like features.

### 2.2. Detection and Localization of mUAVs with Computer Vision

With advances in computational power, vision has become a feasible modality for several tasks with mUAVs. These include fault detection [38], target detection [39] and tracking [40], surveillance [41,42], environmental sensing [43], state estimation and visual navigation [44,45,46,47,48,49], usually combined with other sensors, such as GPS, an inertial measurement unit (IMU), an altimeter or a magnetometer.

Recently, vision has been used for mUAV detection and localization by recognizing black-and-white special markers placed on mUAVs [50,51]. In these studies, circular black-white patterns are designed and used for detection and distance estimation, achieving estimation errors less than 10 cm in real time. However, in some applications where it is difficult to place markers on mUAVs, such approaches are not applicable, and a generic vision-based detection system, such as the one proposed in the current article, is required.

In [52], leader-follower formation flight of two quadrotor mUAVs in an outdoor environment is studied. Relative localization is obtained via monocular vision using boosted cascaded classifiers of Haar-like features for detection and Kalman filtering for tracking. In order to estimate distance, they used the width of the leader with the camera model. They tested their vision-based formation algorithm in a simulation and with real mUAVs. Results for only the real-world experiments are provided where the follower tries to keep a 6-m distance from the leader flying up to a speed of 2 m/s. Their results present only the relative distance of the mUAVs during a flight where the distance information is obtained probably (not mentioned clearly) from GPS. Although they claim that the tracking errors converge to zero, their results indicate that the errors always increase while the leader has a forward motion. Only when the leader becomes almost stationary after 35 s of the total 105 s flight do the errors start to decrease.

In [53], the 2D relative pose estimation problem is studied by extending the approach in [52]. Once the mUAV is detected via a cascaded classifier, its contours are extracted, and for these contours, the best matching image from a set of previously collected images for different view angles is determined. Then, the orientation is estimated by computing the best fitting affine transformation via least squares optimization. Their experimental results are not sufficient to deduce the performance of pose estimation. Furthermore, they use the estimated pose to enhance the relative distance estimation method applied in [52]. According to the results given for only 50 frames, there seems to be an improvement; however, the error is still very high (up to three meters for a 10-m distance with a variance of 1.01 m), and GPS is taken as the ground truth whose inherent accuracy is actually not very appropriate for such an evaluation.

Both studies [52,53] mentioned above use boosted cascaded classifiers for mUAV detection; however, they provide no analysis about the detection and computational performance of the classifiers. The methods are tested only outdoors, and the results for the tracking and pose estimation are poor for evaluating the performances of the methods. They use Haar-like features directly without any investigation. Moreover, no information is available about the camera and processing hardware used. The detection method is reported to run at 5 Hz.

In [54], the collision detection problem for fixed-winged mUAVs is studied. A morphological filter based on the close-minus-open approach is used for the preprocessing stage. Since morphological filters assume a contrast difference between the object and the background, once the image is preprocessed, the resulting candidate regions should be further inspected to get the final estimation. This is very crucial, as the morphological filters produce a large amount of false positives, which have to be eliminated. For this purpose, they combined the morphological filtering stage with two different temporal filtering techniques, namely, Viterbi based and hidden Markov model (HMM) based. The impact of image jitter and the performance of target detection are analyzed by off-board processing of video images on a graphical processing unit (GPU). For jitter analysis, videos recorded using a stationary camera are used by adding artificial jitter at three increasing levels, low, moderate and extreme. Both temporal filtering techniques demonstrate poor tracking performances in the case of extreme jitter where inter-frame motion is greater than four pixels per frame. Some failure periods are also observed for the HMM filter in the moderate jitter case. Target detection performance experiments are performed on videos captured during three different flights with an onboard camera mounted on a UAV. Two of these include head-on maneuvers, and in the third one, UAVs fly at right angles to each other. A detection distance between 400 and 900 m is reported allowing one to estimate a collision before 8–10 s of the impact.

There are also studies for detecting aircraft via vision [55,56,57]. Although we include mainly the literature proposed for mUAVs in this section, these studies are noteworthy, since they are potentially useful for mUAVs, as long as the size, weight and power (SWaP) constraints of mUAVs are complied with. In [55], aircraft detection under the presence of heavily cluttered background patterns is studied for collision avoidance purposes. They applied a modified version of boosted cascaded classifiers using Haar-like features for detection. Temporal filtering is also integrated with the system to reduce false positives by checking the previous detections around a detection before accepting it as valid. Their method does not estimate the distance. Experimental results presented on videos recorded via a camera mounted on an aircraft and having a collision course and crossing scenarios indicate a detection rate of around 80% with up to 10 false positives per frame. No distance information is available between target and host aircraft. Looking at the images, the distance seems to be on the order of some hundred meters. The performance of the system in close distances is also critical, which is not clearly understood from their experiments. They report that their method has a potential of real-time performance; however, no information is available about the frame size of the images and the processing hardware.

The work in [56,57] presents another approach for aircraft detection for sensing and avoiding purposes. They propose a detection method without distance estimation consisting of three stages, which are: (1) morphological filtering; (2) SVM-based classification of the areas found by Stage 1; and (3) tracking based on the similarity likelihoods of matching candidate detections. They tested their method on videos recorded using stationary cameras of various imaging sensor, lens and resolution options. These videos include aircraft flying only above the horizon; therefore, the background patterns are less challenging than the below horizon case, which is not investigated in the study. A detection rate of 98% at five statute miles with one false positive in every 50 frames is reported with a running time of 0.8 s for a four megapixel frame.

### 2.3. Detection and Localization of mUAVs with Other Modalities

There are many alternative sensing methods that can be used for relative localization among mUAVs. One widely-used approach is the Global Positioning System (GPS): in a cooperative scenario, each mUAV can be equipped with GPS receivers and shares its position with other agents [58]. However, GPS signals could be affected by weather, nearby hills, buildings and trees. The service providers may also put limitations on the availability and accuracy of the GPS signals. Moreover, the accuracy of GPS signals is not sufficient for discriminating between close-by neighboring agents unless a real-time kinematic GPS (RTK-GPS) system is used [59]. However, RTK-GPS systems require additional base station unit(s) located in the working environment.

Alternative to GPS, modalities, such as (1) infrared [60,61,62,63,64,65]; (2) audible sound signals [66,67] and (3) ultrasound signals [68,69,70] can be used; however, they entail certain limitations on the distance between the mUAVs and the environments in which they can perform detection. The infrared tends to be negatively affected by sunlight, hence not very suitable for outdoor applications. Sound can be a good alternative; yet, when there are close-by agents, interference becomes a hindrance for multi-mUAV systems, and audible sound signals are prone to be affected by external sound sources. Multipath signals can disturb the measurements severely. The speed of the sound limits the achievable maximum update rate of the system. Moreover, current ultrasound transducers provide limited transmission and reception beam angles, complicating the design of a system with omni-directional coverage.

An alternative modality commonly used by planes is radio waves (*i.e.*, radar). The limitation with radar, however, is that the hardware is too heavy and expensive to place on an mUAV. Recently, there has been an effort to develop an X-band radar to be used on mUAVs [71,72].

Ultra-wide band (UWB) radio modules, which allow two-way time-of-flight and time-difference-of-arrival measurements, and the signal strength between radio frequency (RF) devices could be thought of as other alternatives. However, both techniques need anchor units placed in the environment. The use of UWB modules without beacon units could be considered an aiding method to enhance the performance of localization systems that depend on other modalities. Signal strength between RF devices does not allow one to design an accurate system due to the uncertainties arising from antenna alignment and the effects of close objects.

### 2.4. The Current Study

As reviewed above, there is an interest in detection and distance estimation of aerial vehicles via vision for various purposes, such as cooperation and collision avoidance. Table 1 summarizes these studies in terms of various aspects. Looking at this comparison table and the above explanations, our study fills a void with regard to the comprehensive and systematic analysis of cascaded methods with videos, including very complex indoor and outdoor scenes, providing also an accurate distance estimation method.

**Table 1:** Comparison of the studies on the visual detection of aerial vehicles.

Study	Vehicle	Detection Method	Detection Performance	Motion Blur	Training Time	Testing Time	Background Complexity	Environment	Distance Estimation
Lin *et al.*, 2014	mUAV	Boosted cascaded classifiers with Haar-like features	No	No	No	No	Medium	Outdoor	Yes (low accuracy)
Zhang *et al.*, 2014	mUAV	Boosted cascaded classifiers with Haar-like features	No	No	No	No	Medium	Outdoor	Yes (low accuracy)
Petridis *et al.*, 2008	Aircraft	Boosted cascaded classifiers with Haar-like features	Yes	No	No	No	High	Outdoor	No
Dey *et al.*, 2009; 2011	Aircraft	Morphological filtering	Yes	No	NA	No	Low	Outdoor	No
Lai *et al.*, 2011	mUAV	Morphological filtering	Yes	Yes	NA	Yes	High	Outdoor	No
**Current study**	mUAV	Boosted cascaded classifiers with Haar-like, LBP and HOG features	Yes	Yes	Yes	Yes	High	Indoor and Outdoor	Yes

The main contribution of the article is a systematic analysis of whether an mUAV can be detected using a generic vision system under different motion patterns both indoors and outdoors. The tested indoor motion types include lateral, up-down, rotational and approach-leave motions that are precisely controlled using a physical platform that we constructed for the article. In the outdoor experiments, we tested both calm and agile motions that can also include a moving background. Moreover, the effect of motion blur is also analyzed in a controlled manner. To the best of our knowledge, this is the first study that presents a comprehensive and systematical investigation of vision for detection and distance estimation of mUAVs without special requirements, e.g., the markers used by [50,51].

Besides detecting the quadrotor, our study also integrates a distance estimation method in which a support vector regressor estimates the distance of the quadrotor utilizing the dimensions of the bounding box estimated in the detection phase.

Since it is faster than the alternatives and does not require a large training set, we use cascaded classifiers for detection, which consist of multiple (classification) stages with different complexities [10,34,36,37]. The early (lower) stages of the classifier perform very basic checks to eliminate irrelevant windows with very low computational complexity. The windows passing the lower stages are low in number and undergo heavier computations to be classified as mUAV or background. In order to train a cascaded classifier, we use different feature types proposed in the literature and compare their performances.

## 3. Methods

In this section, we describe the cascaded detection methods used in this paper; namely, the method of Viola and Jones [10,34] and the ones that extend it [36,37].

### 3.1. A Cascaded Approach to mUAV Detection

**Figure 1:**
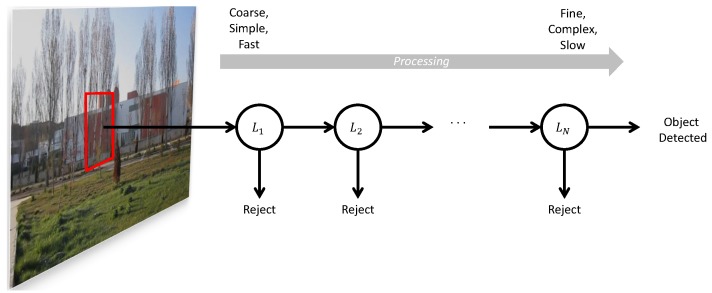
The stages of processing in a cascaded approach. At each stage, a decision to reject or to continue processing is made. If all stages pass, then the method declares detection of the object.

Cascaded classifiers are composed of multiple stages with different processing complexities [10,34,73]. Instead of one highly complex single processing stage, cascaded classifiers incorporate multiple stages with increasing complexities, as shown in Figure 1.

The early stages of the classifier have lower computational complexities and are applied to the image to prune most of the search space quickly. The regions classified as mUAV by one stage of the classifier are passed to the higher stages. As the higher level of stages are applied, the classifier works on a smaller number of regions at each stage to identify them as mUAV or background. At the end of the last stage, the classifier returns the regions classified as mUAV.

In the method proposed by [10,34], which relies on using AdaBoost learning, combinations of weak classifiers are used at each stage to capture an aspect of the problem to be learned. A weak classifier, $h_{f}\left(x\right)$, simply learns a linear classification for feature *f* with a threshold $\theta_{f}$:

(1) $$h_{f}\left(x\right)=\left\{\begin{array}{cc} 1 & \mathrm{if}pf\left(x\right)<p\theta_{f}\\ 0 & \text{otherwise} \end{array}\right.$$

where *p* is the polarity indicating the inequality direction. The best performing weak classifiers are combined linearly to derive a stronger one (at a stage of the cascade); see Algorithm 1.


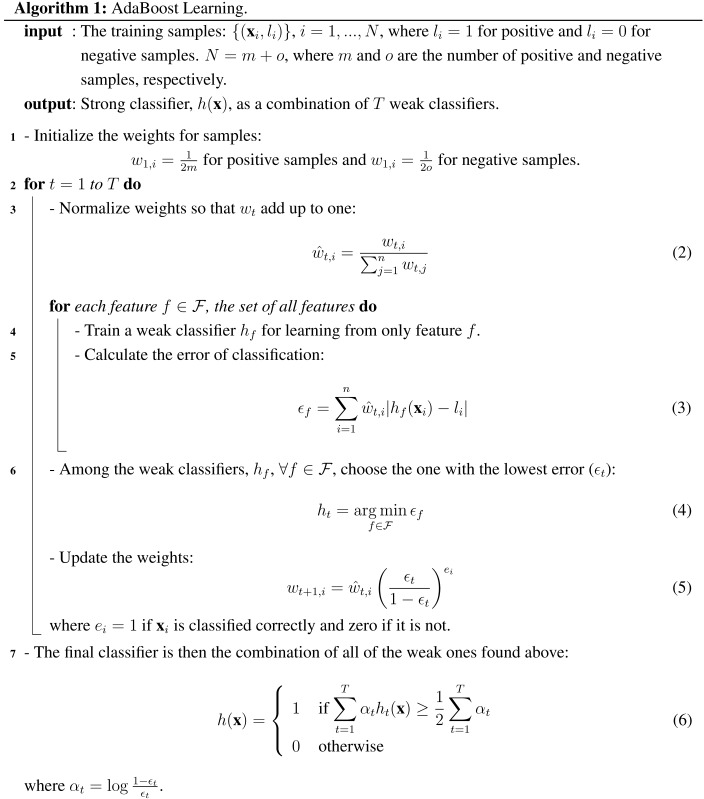


In the approach of Viola and Jones [10,34], the AdaBoost algorithm is used to learn only one stage of the cascade of classifiers: in the cascade, simpler features are used in the earlier stages, whereas bigger and more complex features are only processed if the candidate window passes the earlier stages. The method constructs the cascade by simply adding a new stage of the AdaBoost classifier when the current cascade does not yield the desired false positive and detection rates; see Algorithm 2 and Figure 1. 

**Algorithm 2:** Learning A Cascade of Classifiers (adapted from [34]).
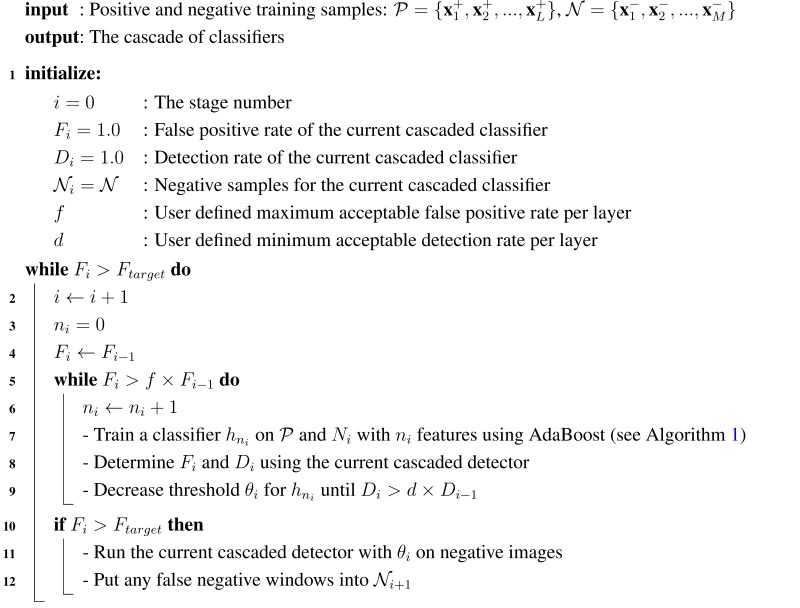


Such an approach can only become computationally tractable if the features can be extracted in a very fast manner. One solution is using integral images, as proposed by Viola and Jones. In Section 3.1.1, we will describe them.

The cascaded detectors are usually run in multiple scales and locations, which lead to multiple detections for the same object. These are merged by looking at the amount of overlap between detections, as a post-processing stage.

#### 3.1.1. Integral Images

In order to speed up the processing, the computation of each feature in a window is performed using the integral images technique. In this method, for a pixel (i,j), the intensities of all pixels that have a smaller row and column number are accumulated at (i,j):

(7) $$II\left(i,j\right)=\sum_{c=1}^{i}\sum_{r=1}^{j}I\left(c,r\right)$$

where *I* is the original image and II the integral image. Note that II can be calculated incrementally from the II of the neighboring pixels more efficiently.

**Figure 2:**
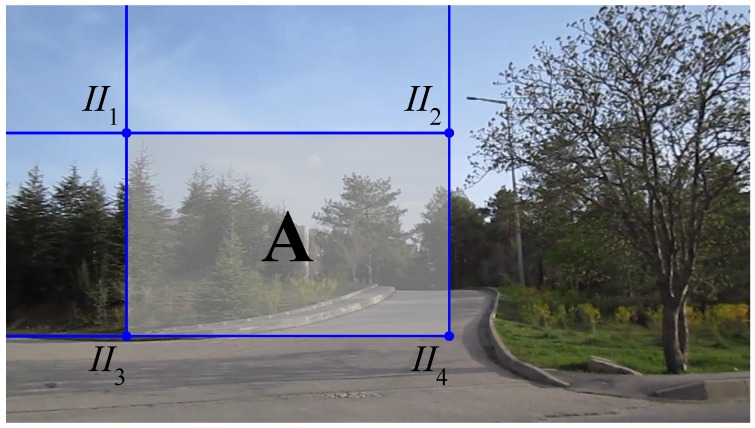
The method of integral images for the efficient computation of sums of intensities in a window. The sum of intensities in Window A can be calculated as $II_{4}+II_{1}-\left(II_{2}+II_{3}\right)$.

Given such an integral image, the sum of intensities in a rectangular window can be calculated easily by accessing four values. See Figure 2 for an example: the sum of intensities in Window A can be calculated as $II_{4}+II_{1}-\left(II_{2}+II_{3}\right)$ [10].

### 3.2. Cascaded Detection Using Haar-like Features (C-HAAR)

**Figure 3:**
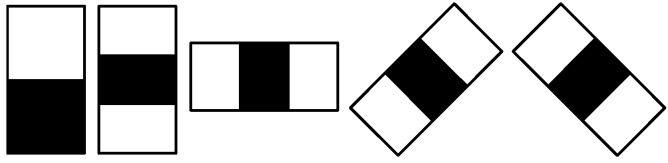
Sample Haar-like features used in our study.

Haar-like features [74] are extensions of Haar wavelets to images. They can be used to extract meaningful information about the distribution of intensities in the form of various configurations of ON and OFF regions in an image window, as shown in Figure 3. Combined with integral images, calculating the responses of Haar-like features at a pixel can be extremely sped up, making it a suitable candidate for the cascaded approach.

In this paper, we are using the extended set of Haar-like features described in [73]. The detector window is run over the image at multiple scales and locations.

### 3.3. Cascaded Detection Using Local Binary Patterns (C-LBP)

In LBP [75], a widely-used method for feature extraction, a window is placed on each pixel in the image within which the intensity of the center pixel is compared against the intensities of the neighboring pixels. During this comparison, larger intensity values are taken as one and smaller values as zero. To describe it formally, for a window $\Omega (x_{c},y_{c})$ at pixel $\left(x_{c},y_{c}\right)$ in image *I*, LBP pattern $L_{p}$ is as $L_{p}\left(x_{c},y_{c}\right)={⊗}_{\left(x,y\right)\in \Omega \left(x_{c},y_{c}\right)}\sigma \left(I\left(x,y\right)-I\left(x_{c},y_{c}\right)\right)$, where ⊗ is the concatenation operator and σ(.) is the unit step function:

(8) $$\sigma \left(x\right)=\left\{\begin{array}{cc} 0 & \mathrm{if}x<0\\ 1 & \text{otherwise} \end{array}\right.$$

The concatenation of ones and zeros can be converted to a decimal number, representing the local intensity distribution around the center pixel with a single number:

(9) $$L_{2}\left(x_{c},y_{c}\right)=\sum_{i=0}^{\left|\Omega (x_{c},y_{c})\right|}2^{i}\times L_{p}^{i}\left(x_{c},y_{c}\right)$$

**Figure 4:**
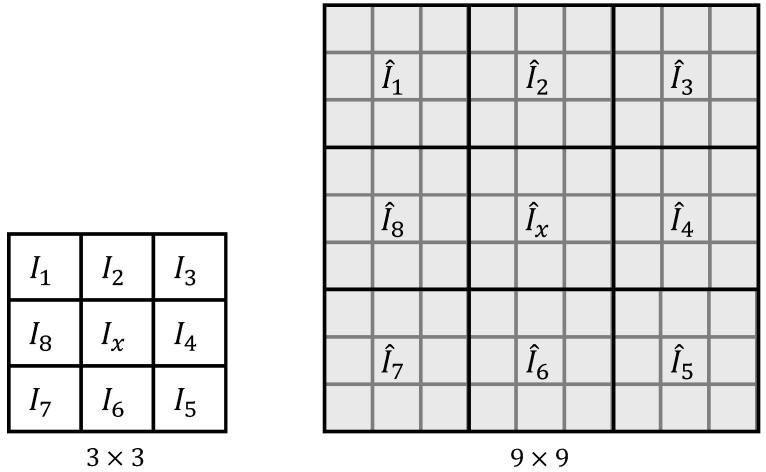
In LBP, the center pixel is compared to the others usually in a 3×3 window (**Left**). In the multi-block version (**Right**), average intensities in the blocks are compared instead.

The cascaded approach of Viola and Jones [10,34] has been extended by Liao *et al.* [36] to use a statistically-effective multi-scale version of LBP (SEMB-LBP) features. In multi-scale LBP, instead of comparing the intensities of pixels, the average intensities of blocks in the window are compared to the central block; see Figure 4. Then, SEMB-LBP at scale *s* is defined as follows:

(10) $$SEMB-LBP_{s}=\left\{\iota \;|\;rank\left(H_{s}\left(\iota \right)\right)<N\right\}$$

where $rank(H_{s})$ is the rank of $H_{s}$ after a descending sort; *N* is the number of uniform patterns, *i.e.*, LBP binary strings where there are at most two 0→1 or 1→0 transitions in the string; and $H_{s}$ is the histogram at scale *s*:

(11) $$H_{s}\left(\iota \right)=1_{\left[f_{s}\left(x,y\right)=\iota \right]},\;\;\iota =0,...,L-1$$

where $f_{s}\left(x,y\right)$ is the outcome of the multi-scale LBP at pixel (x,y). In the current article, we test C-LBP with scales (3×u,3×v), where u=1,...,13 and v=1,...,7, and *N* is set to 63, as suggested by [36]. In order to speed up the computation, the integral image method is used on each bin of the histogram.

### 3.4. Cascaded Detection Using Histogram of Oriented Gradients (C-HOG)

Histogram of oriented gradients (HOG) computes a histogram of gradient occurrences in local grid cells [11]. HOG has been demonstrated to be very successful in human detection and tracking. HOG of an image patch *P* is defined as follows:

(12) $$HOG\left(k\right)=\sum_{p\in P}\delta \left(\;\left\lceil \frac{\theta^{p}}{L}\right\rceil -k\right)$$

where δ(·) is the Kronecker delta which evaluates to one if and only if its input is zero, *L* is a normalizing constant and $\theta^{p}$ is the image gradient orientation at point *p*. HOG(k) is the value of the *k*-th bin in a *K*-bin histogram. In the experiments, we set *K* to 9 which makes the value of *L* equal to 180/K=20 [11].

Zhu *et al.* [37] extended HOG features so that the features are extracted at multiple sizes of blocks at different locations and aspect ratios. This extension enables the definition of an increased number of blocks on which AdaBoost-based cascaded classification (Section 3.1) can be applied to choose the best combination. The integral image method is used on each bin of the histogram to speed up the computation.

### 3.5. Distance Estimation

Having detected the rectangle bounding an mUAV using one of the cascaded approaches introduced above, we can estimate its distance to the camera using the geometric cues. For this, we collect a training set of $\left\{\left(w_{i},h_{i}\right),d_{i}\right\}$, where $w_{i},h_{i}$ are the width and the height of the mUAV bounding box, respectively, and $d_{i}$ is the known distance of the mUAV. Having such a training set, we train a support vector regressor (SVR [76]). Using the trained SVR, we can estimate the distance of the mUAV once its bounding box is estimated.

## 4. Experimental Setup and Data Collection

**Figure 5:**
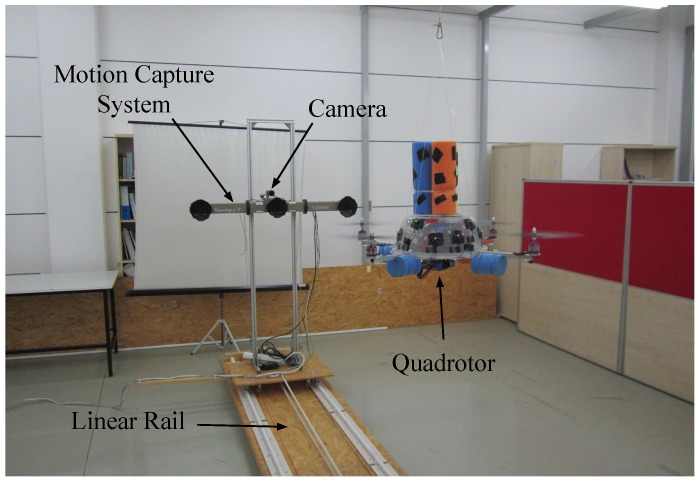
The setup used in indoor experiments. The rail was constructed in order to be able to move the camera with respect to the quadrotor in a controlled manner. This allows analyzing the performance of the methods under different motion types.

**Figure 6:**
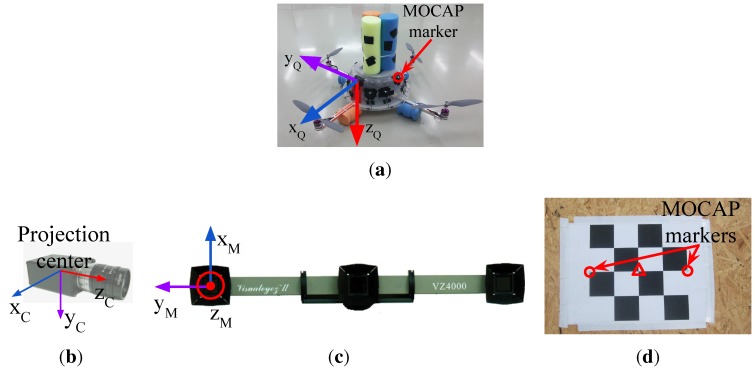
(**a**) The quadrotor used in our study and its body coordinate frame. There are 12 markers mounted roughly $30^{\circ }$ apart from each other on the plastic cup of the quadrotor; (**b**) The body coordinate frame of the camera is defined at the projection center; (**c**) The ${\mathrm{Visualeyez}}^{™}$ II VZ4000 motion capture system and its body coordinate frame; (**d**) The calibration tool used to obtain 3D-2D correspondence points needed to estimate the transformation matrix, $T_{M}^{C}$, between the motion capture system (MOCAP) and the camera coordinate systems. Circles and the triangle indicate the MOCAP markers and the center of the chess pattern, respectively.

The experimental setup, shown in Figure 5, consists of the following components:

- mUAV: We used a quadrotor platform shown in Figure 6a. Open-source Arducopter [77] hardware and software are used as the flight controller. The distance between the motors on the same axis is 60 cm. Twelve markers are placed around the plastic cup of the quadrotor for which we define a rigid body. The body coordinate frame of the quadrotor is illustrated in Figure 6a. The $x_{Q}$-axis and $y_{Q}$-axis are towards the forward and right direction of the quadrotor, respectively. The $z_{Q}$-axis points downwards with respect to the quadrotor.
- Camera: We use two different electro-optic cameras for indoors and outdoors due to varying needs in both environments. For indoors, the synchronization property of the camera is vital, since we have to ensure that the 3D position data obtained from the motion capture system and the captured frames are synchronized in time. Complying with this requirement, we use a camera from Basler ${\mathrm{Scout}}^{™}$ (capturing 1032×778 resolution videos at 30 fps in gray scale) mounted on top of the motion capture system. It weighs about 220 g, including its lens, whose maximum horizontal and vertical angle of views are $93.6^{\circ }$ and $68.9^{\circ }$, respectively. The power consumption of the camera is about 3 W, and it outputs the data through a Gigabit Ethernet port. The body coordinate frame of the camera is centered at the projection center. The $x_{C}$-axis is towards the right side of the camera; the $y_{C}$-axis points down from the camera; and the $z_{C}$-axis coincides with the optical axis of the camera lens, as depicted in Figure 6b.
  Due to difficulties in powering and recording of the indoor camera outdoors, we use another camera (${\mathrm{Canon}}^{®}$ PowerShot A2200 HD) to capture outdoor videos. This camera is able to record videos at a 1280×720 resolution at 30 fps in color. However, we use gray scale versions of the videos in our study.
  Although we needed to utilize a different camera outdoors due to logistic issues, we should note that our indoor camera is suitable to be placed on mUAVs in terms of SWaP constraints. Moreover, alternative cameras with similar image qualities compared to our cameras are also available on the market, even with less SWaP requirements.
- Motion capture system (used for indoor analysis): We use the ${\mathrm{Visualeyez}}^{™}$ II VZ4000 3D real-time motion capture system (MOCAP) (PhoeniX Technologies Incorporated) that can sense the 3D positions of active markers up to a rate of 4348 real-time 3D data points per second with an accuracy of 0.5∼0.7 mm RMS in ∼190 cubic meters of space. In our setup, the MOCAP provides the ground truth 3D positions of the markers mounted on the quadrotor. The system provides the 3D data as labeled with the unique IDs of the markers. It has an operating angle of $90^{\circ }\;\left(\pm 45^{\circ }\right)$ in both pitch and yaw, and its maximum sensing distance is 7 m at minimum exposure. The body coordinate frame of the MOCAP is illustrated in Figure 6c.
- Linear rail platform (used for indoor analysis): We constructed a linear motorized rail platform to move the camera and the MOCAP together in a controlled manner, so that we are able to capture videos of the quadrotor only with single motion types, *i.e.*, lateral, up-down, rotational and approach-leave motions. With this platform, we are able to move the camera and MOCAP assembly on a horizontal line of approximately 5 m up to a 1-m/s speed.

### 4.1. Ground Truth Extraction

In the indoor experimental setup, the MOCAP captures the motion of active markers mounted on the quadrotor and supplies the ground truth 3D positions of those markers. For our purposes, we need the ground truth bounding box of the quadrotor and the distance between the quadrotor and the camera for each frame.

In order to determine a rectangular ground truth bounding box encapsulating the quadrotor in an image, we need to find a set of 2D pixel points ($P_{Qi}^{\prime }$) on the boundaries of the quadrotor in the image (In our derivations, all points in 2D and 3D sets are represented by homogeneous coordinate vectors). These 2D points correspond to a set of 3D points ($P_{Qi}$) on the quadrotor. In order to find $P_{Qi}^{\prime }$, $P_{Qi}$ should first be transformed from the body coordinate frame of the quadrotor to the MOCAP coordinate frame, followed by a transformation to the camera coordinate frame. These two transformations are represented by the transformation matrices $T_{Q}^{M}$ and $T_{M}^{C}$, respectively, and are applied as follows:

(13) $$\begin{array}{c} P_{Mi}=T_{Q}^{M}P_{Qi}\mathrm{for}\mathrm{all}i \end{array}$$

(14) $$\begin{array}{c} P_{Ci}=T_{M}^{C}P_{Mi}\mathrm{for}\mathrm{all}i \end{array}$$

where $P_{Mi}$ and $P_{Ci}$ are the transformed coordinates in the MOCAP and the camera coordinate frames, respectively. After these transformations, we project the points in $P_{Ci}$ to the image plane as:

(15) $$\begin{array}{c} P_{Qi}^{\prime }=P_{c}P_{Ci}\mathrm{for}\mathrm{all}i \end{array}$$

where $P_{c}$ is the camera matrix and get $P_{Qi}^{\prime }$. Then, we can find the bounding box of the quadrotor by calculating the rectangle with the minimum size covering all of the points in $P_{Qi}^{\prime }$ as follows:

(16) $$\begin{array}{cc} x_{r} & =\min(x_{i}) \end{array}$$

(17) $$\begin{array}{cc} y_{r} & =\min(y_{i}) \end{array}$$

(18) $$\begin{array}{cc} w_{r} & =\max\left(x_{i}\right)-\min\left(x_{i}\right) \end{array}$$

(19) $$\begin{array}{cc} h_{r} & =\max\left(y_{i}\right)-\min\left(y_{i}\right) \end{array}$$

where $\left(x_{i},y_{i}\right)\in P_{Qi}^{\prime }$, $\left(x_{r},y_{r}\right)$ is the upper left pixel position of the rectangle and $w_{r}$ and $h_{r}$ are the width and height of the rectangle, respectively.

It is not possible to place a marker on the quadrotor for every point in $P_{Qi}$. Therefore, we define a rigid body, a set of 3D points whose relative positions are fixed and remain unchanged under motion, for 12 markers on the quadrotor. The points in $P_{Qi}$ are then defined virtually as additional points of the rigid body.

A rigid body can be defined from the positions of all markers obtained at a particular time instant while the quadrotor is stationary. However, we wanted to obtain a more accurate rigid body and used the method presented in [78,79] with multiple captures of the marker positions. Taking 60 different samples, we performed the following optimization to minimize the spatial distances between the measured points $M_{i}$ and the points $R_{i}$ in the rigid body model.

(20) $$\underset{R_{i}}{arg min}\sum_{i}{∥M_{i}-R_{i}∥}^{2}$$

where ∥.∥ denotes the calculation of the Euclidean norm for the given vector.

Once the rigid body is defined for the markers on the quadrotor, if at least four markers are sensed by the MOCAP, $T_{Q}^{M}$ can be estimated. Since the MOCAP supplies the 3D position data as labeled and the rigid body is already defined using these labels, there is no correspondence matching problem. Finding such a rigid transformation between two labeled 3D point sets requires the least squares fitting of these two sets and is known as the “absolute orientation problem” [80]. We use the method presented in [78,81] to solve this problem and calculate $T_{Q}^{M}$. Note that the $T_{Q}^{M}$ transformation matrix should be calculated whenever the quadrotor and the camera moves with respect to each other.

There is no direct way of calculating $T_{M}^{C}$, since it is not trivial to measure the distances and the angles between the body coordinate frames of the MOCAP and the camera. However, if we know a set of 3D points ($P_{Ti}$) in the MOCAP coordinate frame and a set of 2D points ($P_{Ti}^{\prime }$) which corresponds to the projected pixel coordinates of the points in $P_{Ti}$, then we can estimate $T_{M}^{C}$ as the transformation matrix that minimizes the re-projection error. The re-projection error is given by the sum of squared distances between the pixel points in $P_{Ti}^{\prime }$ as in the following optimization criterion:

(21) $$\underset{T_{M}^{C}}{arg min}\sum_{i}{∥P_{Ti}^{\prime }-T_{M}^{C}P_{Ti}∥}^{2}$$

For collecting the data points in $P_{Ti}$ and $P_{Ti}^{\prime }$, we prepared a simple calibration tool shown in Figure 6d. In this tool, there is a chess pattern and 2 MOCAP markers mounted on the two edges of the chess pattern. The 3D position of the chess pattern center, shown inside the triangle in Figure 6d, is calculated by finding the geometric center of the marker positions. We obtain the 2D pixel position of the chess pattern center using the camera calibration tools of the Open Source Computer Vision Library (OpenCV) [82]. We collect the data needed for $P_{Ti}$ and $P_{Ti}^{\prime }$ by moving the tool in front of the camera. Note that, since the MOCAP and the camera are attached to each other rigidly, once $T_{M}^{C}$ is estimated, it is valid as long as the MOCAP and the camera assembly remain fixed.

In order to calculate the ground truth distance between the quadrotor and the camera, we use $T_{Q}^{M}$ and $T_{M}^{C}$ as follows:

(22) $$\begin{array}{c} p_{c}^{\prime }=T_{M}^{C}T_{Q}^{M}p_{c} \end{array}$$

where $p_{c}$ is the 3D position of the quadrotor center in the quadrotor coordinate frame and $p_{c}^{\prime }$ is the transformed coordinates of the quadrotor center to the camera coordinate frame. $p_{c}$ is defined as the geometric center of 4 points where the motor shafts and the corresponding propellers intersect. Once $p_{c}^{\prime }$ is calculated, the distance of the quadrotor to the camera ($d_{Q}$) is calculated as:

(23) $$\begin{array}{c} d_{Q}=∥p_{c}^{\prime }∥ \end{array}$$

### 4.2. Data Collection for Training

**Figure 7:**
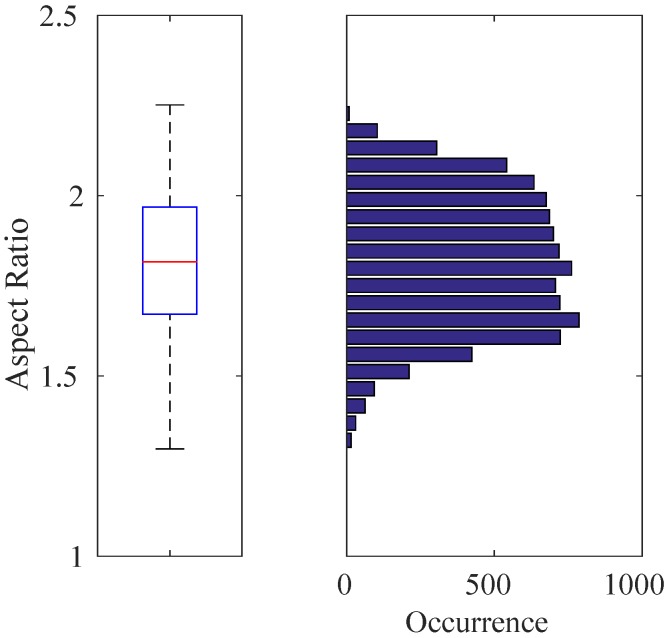
Box-plot (**Left**) and histogram (**Right**) representation for the aspect ratios of 8876 quadrotor images automatically extracted from the training videos. In this figure and the subsequent box-plot figures, the top and bottom edges of the box and the line inside the box represent the first and third quartiles and the median value, respectively. The bottom and top whiskers correspond to the smallest and largest non-outlier data, respectively. The data inside the box lie within the 50% confidence interval, while the confidence interval of the data in between the whiskers is 99.3%. Here, the median value is 1.8168, which defines the aspect ratio of the training images used.

**Figure 8:**
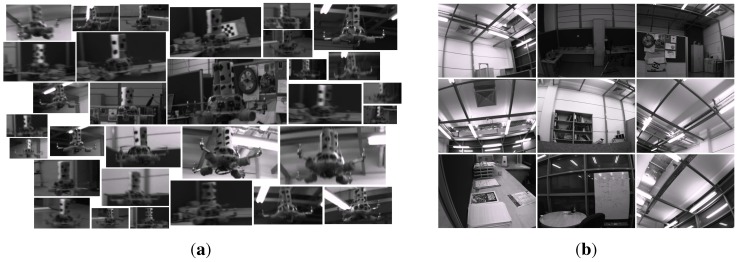
Example images from indoor (**a**) quadrotor and (**b**) background training image sets. Mostly the challenging examples are provided in the quadrotor images.

Indoors: We recorded videos of the quadrotor by moving the MOCAP and the camera assembly around the quadrotor manually while the quadrotor is hanging at different heights from the ground and stationary with its motors running. From these videos, we automatically extracted 8876 image patches, including only the quadrotor using the bounding box extraction method described in Section 4.1 without considering the aspect ratios of the patches. The distribution of the aspect ratios for these images is given in Figure 7 with a median value of 1.8168. Since the training of cascaded classifiers requires image windows with a fixed aspect ratio, we enlarged the bounding boxes of these 8876 images by increasing their width or height only according to the aspect ratio of the originally extracted image window, so that they all have a fixed aspect ratio of approximately 1.8168 (due to floating point rounding, aspect ratios may not be exactly 1.8168). We preferred enlargement to fix the aspect ratios, since this approach keeps all relevant data of the quadrotor inside the bounding box. We also recorded videos of the indoor laboratory environment without the quadrotor in the scene. From these videos, we extracted 5731 frames at a resolution of 1032×778 pixels as our background training image set. See Figure 8a,b for sample quadrotor and background images captured indoors.

**Figure 9:**
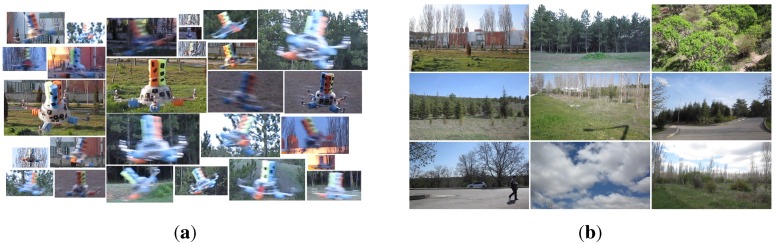
Example images from outdoor (**a**) quadrotor and (**b**) background training image sets. The images are colored; however, their grayscale versions are used in the training. For quadrotor images, mostly the challenging examples are included.

Outdoors: We used a fixed camera to record the quadrotor while it is flying in front of the camera using remote control. Since the MOCAP is not operable outdoors, the ground truth is collected in a labor-extensive manner: by utilizing the background subtraction method presented in [83], we are able to approximate the bounding box of the quadrotor in these videos as long as there are not any moving objects other than the quadrotor. Nevertheless, it is not always possible to get a motionless background. Therefore, the bounding boxes from background subtraction are inspected manually, and only the ones that bound the quadrotor well are selected. Both the number and aspect ratio of the outdoor training images are the same as the indoor images. For outdoor background training images, we have recorded videos at various places on the university campus. These videos include trees, bushes, grass, sky, roads, buildings, cars and pedestrians without the quadrotor. From these videos, we have extracted frames as the same number of indoor background training images at 1280×720 resolution. See Figure 9a,b for sample images collected outdoors.

Looking at the training image sets, the following observations can be deduced, which also represent the challenges in our problem: (i) changes in camera pose or quadrotor pose result in very large differences in the quadrotor’s visual appearance; (ii) the bounding box encapsulating the quadrotor contains a large amount of background patterns due to the structure of the quadrotor; (iii) vibrations in the camera pose and the agile motions of the quadrotor cause motion blur in the images; (iv) changes in brightness and the illumination direction yield very different images; and (v) motion in the image can also be induced by the motion of the camera or the motion of background objects (e.g., trees swinging due to wind, *etc.*).

### 4.3. Data Collection for Testing

Indoor and outdoor environments are significantly different from each other, since controlled experiments can only be performed indoors by means of motion capture systems. On the other hand, outdoor environments provide more space, increasing the maneuverability of the quadrotor and causing many challenges that need to be evaluated. These differences directed us to prepare test videos of different characteristics indoors and outdoors.

**Figure 10:**
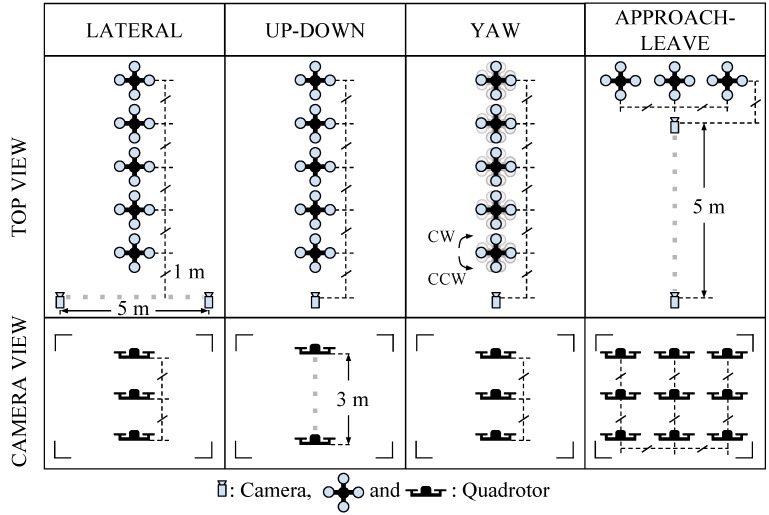
Graphical representation for indoor test videos. There are 4 motion types, namely lateral, up-down, yaw and approach-leave. Each of them is illustrated with the top and camera views. Dashed gray thick lines represent the motion of the camera or the quadrotor along the path with the given length. Dashed black thin lines are used to represent dimensions.

In order to investigate the performance of the methods (C-HAAR, C-LBP and C-HOG) systematically, we defined 4 different motion types, namely lateral, up-down, yaw and approach-leave, for the indoor test videos. Please note that maneuvers in a free flight are combinations of these motions, and use of these primitive motions is for systematic evaluation purposes. The recording procedure of each motion type is depicted in Figure 10 for two different views, the top view and the camera view. Each motion type has different characteristics in terms of the amount of changes in the scale and appearance of the quadrotor, as well as the background objects, as shown in Table 2. The details of each motion type are as follows:

- Lateral: The camera performs left-to-right or right-to-left maneuvers while the quadrotor is fixed at different positions, as illustrated in Figure 10. As seen in the top view, the perpendicular distance of the quadrotor to the camera motion course is changed by 1 m for each of 5 distances. For each distance, the height of the quadrotor is adjusted to 3 different (top, middle and bottom) levels with 1 m apart, making a total of 15 different position for lateral videos. Left-to-right and right-to-left videos collected in this manner allow us to test the features’ resilience against large background changes.
  In each video, the camera is moved along an approximately 5-m path. However, when the perpendicular distance is 1 m and 2 m and, the quadrotor is not fully visible in the videos for the top and bottom levels. Therefore, these videos are excluded from the dataset, resulting in 22 videos with a total of 2543 frames.
- Up-down: The quadrotor performs a vertical motion from the floor to the ceiling for the up motion and *vice versa* for the down motion. The motion of the quadrotor is performed manually with the help of a hanging rope. The change in the height of the quadrotor is approximately 3 m in each video. During the motion of the quadrotor, the camera remains fixed. For each of the 5 different positions shown in Figure 10, one up and one down video are recorded, resulting in 10 videos with a total of 1710 frames. These videos are used for testing the features’ resilience against large appearance changes.
- Yaw: The quadrotor turns around itself in a clockwise or counter clockwise direction, while both the camera and the quadrotor are stationary. The quadrotor is positioned at the same 15 different points used in the lateral videos. Since the quadrotor is not fully present in the videos recorded for the top and bottom levels when the perpendicular distance is 1 m and 2 m, these videos are omitted from the dataset. Hence, there are 22 videos with a total of 8107 frames in this group. These videos are used for testing the features’ resilience against viewpoint changes causing large appearance changes.
- Approach-leave: In these videos, the camera approaches the quadrotor or leaves from it while the quadrotor is stationary. There are 9 different positions for the quadrotor a with 1-m distance separation, as illustrated in Figure 10. The motion path of the camera is approximately 5 m. Approach and leave videos are recorded separately and we have 18 videos with a total of 3574 frames for this group. These videos are used for testing whether the features are affected by large scale and appearance changes.

We should note that the yaw orientation of the quadrotor is set to random values for each of 50 videos in the lateral, up-down and approach-leave sets, although the quadrotors in Figure 10 are given for a fixed orientation. There are cases where the MOCAP can give the wrong or insufficient data to extract the ground truth for some frames. These frames are not included in the dataset.

For outdoor experiments, we prepared four different videos with distinct characteristics. In all videos, the quadrotor is flown manually in front of a stationary camera. In the first two videos, a stationary background is chosen. These two videos differ in terms of agility, such that in the first video, the quadrotor performs calm maneuvers, whereas in the second one, it is flown in an agile manner. In the third video, the background includes moving objects, like cars, motorcycles, bicycles and pedestrians, while the quadrotor is flown in a calm manner. The fourth video is recorded to test the maximum detection distances of the methods. In this video, the quadrotor first leaves from the camera and then comes back, flying on an approximately straight 110-m path. We will call these videos (i) calm, (ii) agile, (iii) moving background and (iv) distance in the rest of the paper. These videos have 2954, 3823, 3900 and 2468 frames, respectively. The ground truth bounding boxes for each frame of calm, agile and moving background videos are extracted manually. For the distance video, only the ground truth distance of the quadrotor to the camera is calculated by utilizing another video recoded simultaneously by a side view camera. With the help of poles at known locations in the experiment area and by manually extracting the center of the quadrotor from the side view video, we computed the ground truth distance with simple geometrical calculations.

**Table 2:** Properties of motion types in terms of the amount of changes in the scale and appearance of the quadrotor and the background objects.

	Lateral	Up-Down	Yaw	Approach-Leave
Scale	Moderate	Moderate	Small	Large
Appearance	Moderate	Large	Large	Large
Background	Large	No Change	No Change	Moderate

We should note that the scenes used in testing videos are different from the ones included in the training datasets for both indoors and outdoors.

Our dataset is available at http://www.kovan.ceng.metu.edu.tr/fatih/sensors/.

## 5. Results

We implemented the cascaded methods introduced in Section 3 using OpenCV [82] and evaluated them on the indoor and outdoor datasets. We trained indoor and outdoor cascade classifiers separately using the corresponding training datasets with the following parameters: The quadrotor image windows were resized to 40×22 pixels. For an image with this window size, C-HAAR extracts 587,408 features, whereas C-LBP and C-HOG yield 20,020 and 20 features, respectively. Then, 7900 positive (quadrotor) and 10,000 negative (background) samples were used for indoors and outdoors. We trained the classifiers with 11, 13, 15, 17 and 19 stages (the upper limit of 19 is due to the enormous time required to train C-HAAR classifiers, as will be presented in Section 5.6.1). During our tests, the classifiers performed multi-scale detections beginning from a minimum window size of 80×44 and enlarging the window size by multiplying it with 1.1 at each scale.

### 5.1. Performance Metrics

In order to evaluate the detection performance of the classifiers, we use precision-recall (PR) curves, which are drawn by changing the threshold of the classifiers’ last stages from -100 to +100, as performed by [10,34]. Note that each stage of the cascaded classifiers has its own threshold determined during the training and that decreasing the threshold of a stage *S* to a low value, such as -100, results in a classifier with S-1 many stages at the default threshold.

Precision is defined as:

(24) $$Precision=\frac{tp}{tp+fp}$$

where tp is the number of true positives (see below) and fp is the number of false positives. Recall is defined as:

(25) $$Recall=\frac{tp}{tp+fn}$$

where fn is the number of false negatives.

A detected bounding box ($B_{D}$) is regarded as a true positive if its Jaccard index (J) [84], calculated as follows, is greater than 60%:

(26) $$J\left(B_{D},B_{G}\right)=\frac{|B_{D}\cap B_{G}|}{|B_{D}\cup B_{G}|}$$

where $B_{G}$ is the ground truth bounding box. Otherwise, $B_{D}$ is regarded as a false positive. If there are multiple detections in a frame, each $B_{D}$ is evaluated separately as a tp or fp. If no $B_{D}$ is found for an image frame by the classifier, then fn is incremented by one.

We use also F-score in our evaluations, calculated as follows:

(27) $$F-Score=2\times \frac{Precision\times Recall}{Precision+Recall}$$

A widely-used measure with PR-curves is the normalized area under the curve. If a PR curve, p(x), is defined at the interval $\left[r_{min},r_{max}\right]$, where $r_{min}$ and $r_{max}$ are the minimum and maximum recall values, respectively, the normalized area $A_{p}$ under curve p(x) is defined as:

(28) $$A_{p}=\frac{1}{r_{max}-r_{min}}\int_{r_{min}}^{r_{max}}p\left(x\right)\;dx$$

### 5.2. Indoor Evaluation

**Figure 11:**
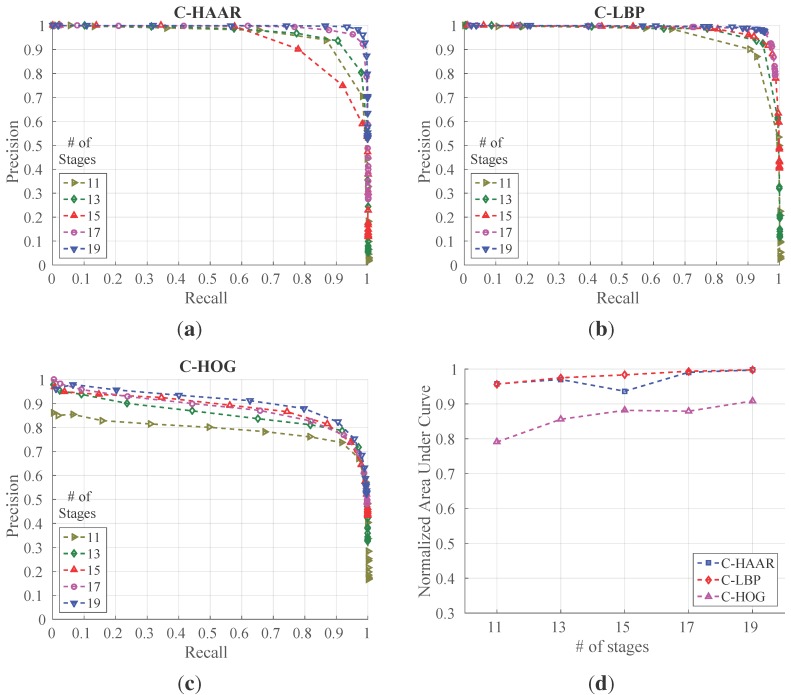
Precision-recall (PR) curves showing the performance of (**a**) C-HAAR, (**b**) C-LBP and (**c**) C-HOG for different numbers of stages on indoor test videos; (**d**) normalized areas under the PR curves in (**a**–**c**).

We tested the classifiers trained with the indoor training dataset on indoor test videos having 15,934 frames in total with four different motion types, namely lateral, up-down, yaw and approach-leave, as presented in Section 4.3. We evaluated the classifiers for five different numbers of stages to understand how they perform while their complexity increases. Figure 11 shows the PR curves, as well as the normalized area under the PR curves for each method and for different numbers of stages. In Table 3, the maximum F-score values and the values at default thresholds are listed.

**Table 3:** Performance of the methods indoors, reported as F-score values. Bold indicates best performances.

Feature Type	C-HAAR					C-LBP					C-HOG				
Number of Stages	11	13	15	17	19	11	13	15	17	19	11	13	15	17	19
Maximum F-Score	0.903	0.920	0.836	0.958	**0.976**	0.904	0.936	0.940	0.962	**0.964**	0.818	0.848	0.842	0.839	**0.862**
F-Score at Default Threshold	0.058	0.143	0.286	0.570	**0.822**	0.104	0.345	0.774	0.943	**0.954**	0.404	0.550	0.627	0.664	**0.716**

The performances of C-HAAR and C-LBP are close to each other in terms of maximum F-scores (Table 3) and the normalized area under the curve (Figure 11d), except for a decrease at Stage 15 of C-HAAR, and they both perform better than C-HOG in all aspects. The lower performance of C-HOG is due to the low number of features it extracts from a training window. Even with the extension of Zhu *et al.* [37], only 20 features are extracted from a 40×22-${\mathrm{pixel}}^{2}$ training image. For AdaBoost to estimate a better decision boundary, more features are required. The difference between the number of features used by C-HAAR and C-LBP, however, does not result in a considerable performance divergence.

We observe a slight difference between C-HAAR and C-LBP in terms of the lowest points that PR curves (Figure 11) reach. This is related to the performance differences between the methods at their default threshold. As mentioned earlier, decreasing the threshold of a classifier’s latest stage, *S*, to a very low value results in a classifier with a stage number of S-1. Therefore, since the performances of C-LBP classifiers at their default thresholds are greater than the default performances of C-HAAR classifiers, we observe PR curves ending at higher points in the case of C-LBP.

**Figure 12:**
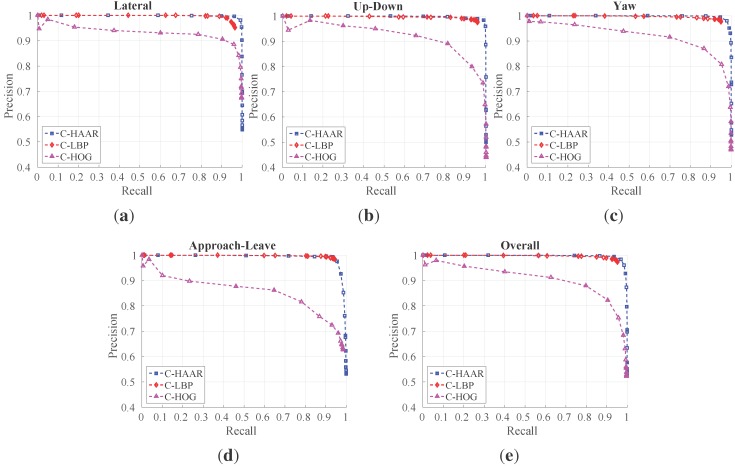
PR curves for: (**a**) lateral left-to-right and right-to-left; (**b**) up and down; (**c**) yaw clockwise and counter-clockwise; (**d**) approach and leave; and (**e**) all motion types.

For all methods, training with 19 stages outperforms training with less stages. Therefore, taking 19 as the best stage number for all methods, we present their performances on different motion types in Figure 12 with their overall performances on all motion types. The performance of C-HAAR is slightly better than C-LBP on lateral, up-down and yaw motions, since it has PR curves closer to the rightmost top corner of the figures. C-HOG gives the worst performance in all motion types.

When we look at the performances of each method individually for each motion type, C-HAAR performs similar on lateral, up-down and yaw motions; however, its performance diminishes on approach-leave, which is the most challenging motion in the indoor dataset. C-LBP has performance degradation on lateral motion, showing that it is slightly affected by the large background changes. Other than this, the performance of C-LBP is almost equal for other motion types. C-HOG performs better on lateral than other motions. Notable performance degradation is observed for the approach-leave motion.

### 5.3. Outdoor Evaluation

**Figure 13:**
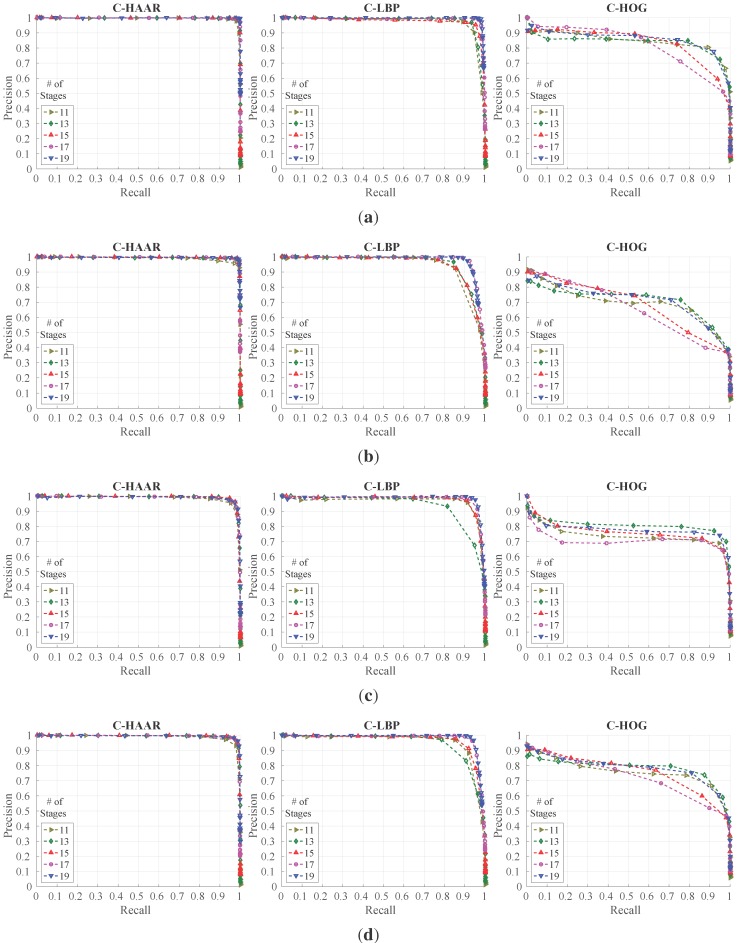
PR curves for outdoor evaluation (best viewed in color). (**a**) Performances for calm test video; (**b**) performances for agile test video; (**c**) performances for moving background test video; (**d**) overall performances.

**Figure 14:**
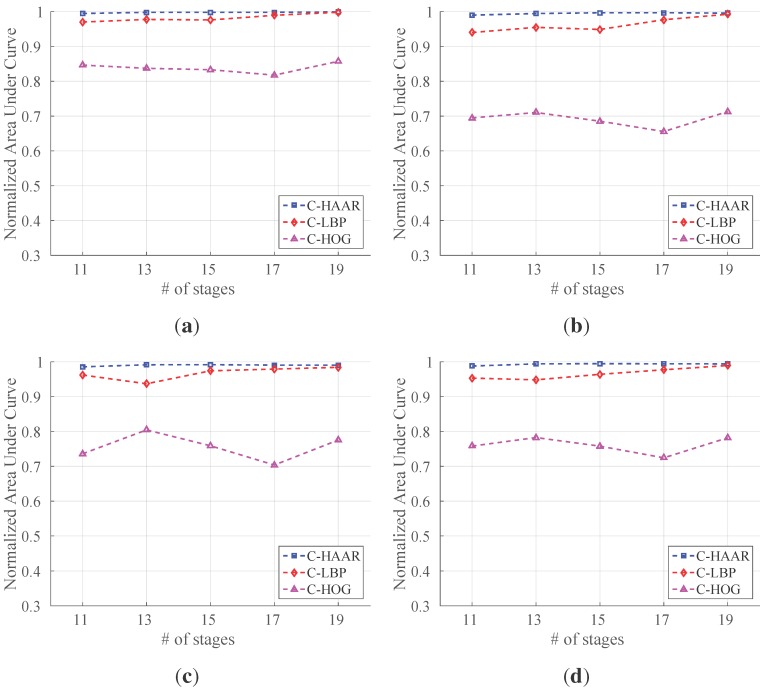
Normalized area under the curves for outdoor evaluation. (**a**) Stationary background calm flight; (**b**) stationary background agile flight; (**c**) moving background calm flight; (**d**) all outdoor flights combined.

We evaluated the classifiers trained with the outdoor training dataset using all outdoor motion types, namely calm, agile and moving background. For each motion type and for overall performance, we present the resulting PR curves and the normalized area under the curves in Figure 13 and Figure 14, respectively. The F-score performances are listed in Table 4.

We notice that the performances of C-HAAR and C-LBP are remarkably better than C-HOG in all experiments. When comparing C-HAAR and C-LBP, C-HAAR gives slightly better results in terms of all measures. Under the agile maneuvers of the quadrotor, C-LBP and C-HOG display performance degradation, while C-HAAR’s performance is hardly affected. This suggests that C-HAAR is more robust against appearance changes due to the rotation of the quadrotor. Slight performance decreases are observed in moving background video for C-HAAR and C-LBP.

When compared to the indoor evaluation, C-HAAR classifiers with low stage numbers perform better outdoors. The performance of C-HOG decreases in outdoor tests. In terms of the F-score, the best performing stage numbers differ for C-HAAR and C-HOG. Unlike indoors, the performances of the C-LBP and C-HAAR classifiers at their default thresholds are close to each other, resulting in PR curves reaching closer end points when compared to indoor results.

In order to determine the maximum distances at which the classifiers can detect the quadrotor successfully, an experiment is conducted with distance test video using the best performing classifiers overall according to the F-scores in Table 4. In this experiment, the minimum detection window size is set to 20×11. The resulting maximum detection distances are 25.71 m, 15.73 m and 24.19 m, respectively, for C-HAAR, C-LBP and C-HOG.

**Table 4:** Performance of the methods outdoors, reported as F-score values. Bold indicates best performances.

	Feature Type	C-HAAR					C-LBP					C-HOG				
	Number of Stages	11	13	15	17	19	11	13	15	17	19	11	13	15	17	19
CALM	Maximum F-Score	0.979	0.987	0.991	0.991	**0.997**	0.930	0.951	0.953	0.977	**0.985**	**0.846**	0.822	0.781	0.732	0.842
	F-Score at Default Threshold	0.036	0.112	0.248	0.536	**0.734**	0.040	0.095	0.266	0.670	**0.930**	**0.118**	0.144	0.168	0.189	0.216
AGILE	Maximum F-Score	0.965	0.983	0.988	0.987	**0.989**	0.887	0.902	0.890	**0.947**	0.942	0.719	**0.735**	0.619	0.600	0.713
	F-Score at Default Threshold	0.034	0.108	0.282	0.727	**0.906**	0.041	0.094	0.260	**0.704**	0.920	0.121	**0.146**	0.168	0.188	0.211
MOVING BACKGROUND	Maximum F-Score	0.955	0.965	**0.969**	0.963	0.967	0.935	0.870	0.940	0.954	**0.964**	0.797	**0.840**	0.785	0.777	0.832
	F-Score at Default Threshold	0.030	0.084	**0.169**	0.274	0.441	0.043	0.111	0.269	0.480	**0.747**	0.158	**0.180**	0.199	0.216	0.234
**OVERALL**	Maximum F-Score	0.955	0.972	**0.977**	0.973	0.975	0.906	0.869	0.915	0.949	**0.957**	0.770	**0.801**	0.707	0.672	0.781
	F-Score at Default Threshold	0.033	0.099	**0.221**	0.429	0.627	0.042	0.100	0.265	0.594	**0.850**	0.132	**0.157**	0.178	0.198	0.221

### 5.4. Performance under Motion Blur

We have tested the performance of the methods against motion blur in the images. We utilized a linear motion blur similar to the one used in [85,86]. A motion-blurred version of an image *I* is generated by convolving it with a filter *k* (*i.e.*, $\tilde{I}=I\text{*}k$), which is defined as:

(29) $$k\left(x,y\right)=\left\{\begin{array}{cc} 1 & \mathrm{if}y=d/2\\ 0 & \text{otherwise} \end{array}\right.$$

where *d* is the dimension of the kernel (blur length), determining the amount of motion blur, sampled from a Gaussian distribution N(μ=0,σ), with *μ* and *σ* being the mean and the standard deviation, respectively. We applied this kernel to the video images after a rotation of *θ* radian (blur angle) chosen from a uniform distribution U(0,π). For each frame of a video, a new kernel is generated in this manner, and it is applied to all pixels in that frame. Using this motion blur model, we generated blurred versions of all indoor test videos for five different values of *σ*, namely, 5, 10, 15, 20 and 25.

**Figure 15:**
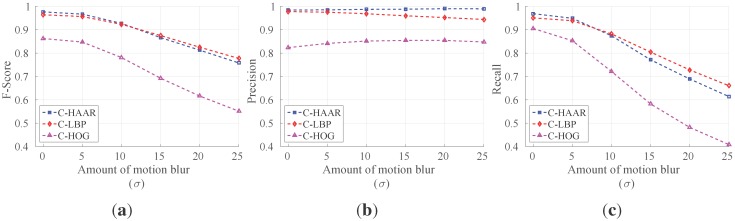
Performance of methods under motion blur. (**a**) F-score; (**b**) precision; and (**c**) recall. σ=0 corresponds to original videos without motion blur.

We tested the best performing classifiers having 19 stages and giving the maximum F-scores in Table 3 on the blurred and original videos. The tests are performed on the indoor dataset only, for the sake of simplicity, since we do not expect a difference between the effects of motion blur for indoors and outdoors. The results depicting the changes in F-score, precision and recall against the amount of motion blur are given in Figure 15. We see that C-HAAR and C-LBP display a more robust behavior compared to C-HOG, since the decreasing trend in their F-score and recall values is slower than C-HOG. C-LBP performs better than C-HAAR in terms of F-score and recall. However, the precision of C-HAAR and C-HOG increases slightly with the increasing amount of motion blur. The reason for this increase is the decrease in the number of false positives, since they start to be identified as background by C-HAAR and C-HOG when there is more noise. However, this trend has a limit, since, at some point, the noise causes a major decrease in the number of true positives. Here, σ=25 is the point where the precision of C-HAAR and C-HOG starts to decrease.

In the case of C-LBP, precision values are continuously decreasing due to an increasing number of false positives. However, this degradation in precision is not so rapid. Moreover, the decreasing trend in the recall of C-LBP is slower than other methods. This slow decline rate in the recall results from a high number of correct detections and a low number of incorrect rejections.

### 5.5. Distance Estimation

In order to train the distance estimator (Section 3.5), we prepared a training set of 35,570 pairs of $\left\{\left(w_{i},h_{i}\right),d_{i}\right\}$, where $w_{i},h_{i}$ are the width and the height of the mUAV bounding box, respectively, and $d_{i}$ is its known distance, acquired using the motion capture system (see Section 4 for the details).

**Figure 16:**
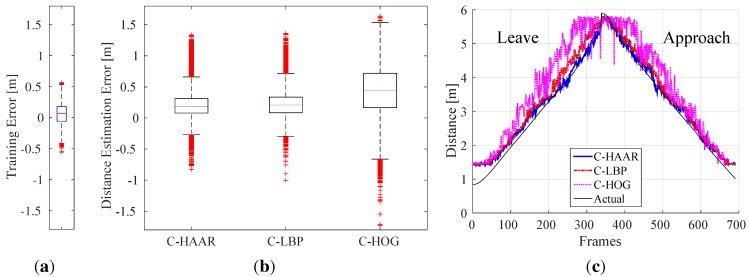
(**a**) Training error distribution for distance estimation; (**b**) distribution of distance estimation error for each method; (**c**) distance estimations during a leaving motion followed by an approach.

A support vector regressor (SVR) has been trained on this set with the radial basis function kernel. The values of the parameters are optimized using a grid-search and five-fold cross-validation, yielding the following values: ν=0.09,C=0.1 and γ=0.00225. With these values, a training error of 6.44 cm as the median is obtained. The distribution of distance estimation errors over the training set is shown in Figure 16a.

Since there is no ground truth distance information at hand for the outdoor dataset, the distance estimation has been evaluated by means of indoor videos only.

As in motion-blur analysis, we tested the best performing classifiers having 19 stages resulting in maximum F-scores tabulated in Table 3. The resulting distance estimation distributions are displayed in Figure 16b.

We see that the performance of C-HAAR is slightly better than C-LBP. The medians of the error for C-HAAR and C-LBP are 18.6 cm and 20.83 cm, respectively. The performance of C-HOG is worse than the other two methods with a median error of 43.89 cm and with errors distributed over a larger span.

In Figure 16c, we plot estimated and actual distances for a leave motion followed by an approach. These plots are consistent with the results provided with Figure 16b, such that the performances of C-HAAR and C-LBP are close to each other and better than C-HOG.

#### 5.5.1. Time to Collision Estimation Analysis

We have analyzed the performance of the methods in the estimation of time to collision (TTC). In order to estimate TTC, the current speed ($v_{c}$) is estimated first:

(30) $$v_{c}=\frac{d_{c}-d_{p}}{\Delta t}$$

where $d_{c}$ is current distance estimation, $d_{p}$ is a previous distance estimation and Δt is the time difference between two distance estimations. $d_{p}$ is arbitrarily selected as the 90th previous distance estimation to ensure a reliable speed estimation. Once $v_{c}$ is calculated, TTC can be estimated as:

(31) $$TTC=\frac{d_{c}}{v_{c}}$$

**Figure 17:**
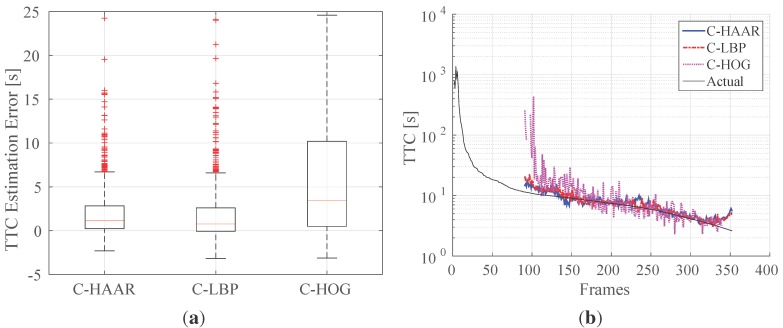
Indoor time to collision estimation performances of the methods for (**a**) all approach motions and (**b**) a single approach motion. In (**a**), there are outliers also outside the limits of the y-axis. However, in order to make differences between the methods observable, y-axis is limited between -5 and 25. In (**b**), the y-axis is in log-scale, and no estimation is available until the 90th frame. The missing points after the 90th frame are due to negative or infinite time to collision estimations.

Using this approach, we have evaluated the methods on indoor approach videos. Figure 17a shows the resulting box-plots for errors in estimating TTC. Figure 17b illustrates the estimated and actual TTC’s for a single approach video. The performances of C-HAAR and C-LBP are close to each other with a smaller median error for C-LBP. C-HOG performs worse than C-HAAR and C-LBP as a result of its low performance in distance estimation.

### 5.6. Time Analysis

The training and testing time of the methods are analyzed in detail for the indoor and outdoor datasets on a computer with an ${\mathrm{Intel}}^{®}$ ${\mathrm{Core}}^{™}$ i7-860 processor clocked at 2.80-GHz and 8 GB DDR3-1333MHz memory, running Ubuntu 14.04. Currently, processors with similar computational power are available for mUAVs [87,88].

#### 5.6.1. Training Time Analysis

**Figure 18:**
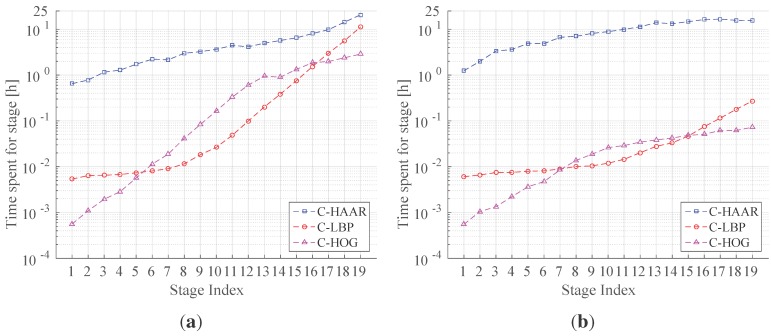
(**a**) Indoor and (**b**) outdoor training times consumed for each stage in the cascaded classifier. The y-axes are in log-scale.

Figure 18 shows the amount of time required to train each stage of the classifiers, and Table 5 lists the total training times needed for the training of all 19 stages (the upper limit of 19 has been imposed due to the excessive time required for training C-HAAR). We observe that C-HAAR is the most time consuming method, which is succeeded by C-LBP and C-HOG. It is observed that C-HAAR requires on the order of days for training, whereas C-LBP and C-HOG finish in less than an hour.

The main reason behind the differences in the training times of the methods is the number of features extracted by each method from an image window. As mentioned previously (Section 5), the ordering among the methods is C-HAAR, C-LBP and C-HOG, with the decreasing number of associated features with an image window of 40×22 pixels. The increase in the number of features amounts to an increase in training the cascaded classifier to select the subset of good features via boosting.

**Table 5:** Time spent for training the cascaded classifiers having 19 stages in hours.

Feature Type	C-HAAR	C-LBP	C-HOG
Indoor	98.31	22.94	13.53
Outdoor	177.59	0.87	0.52

We also observe a significant difference between indoor and outdoor training times for each method. For the outdoor dataset, C-HAAR is twice as slow as for the indoor dataset, where C-LBP and C-HOG are 26-times faster. The reason for this is the fact that the outdoor background images are more distinct, enabling C-LBP and C-HOG to find the best classifier in each stage more quickly. However, this effect is not observed in C-HAAR, since Haar-like features are adversely affected by the illumination changes, which are observed substantially in our outdoor dataset.

#### 5.6.2. Testing Time Analysis

We have measured and analyzed the computation time of each method in two different aspects: (i) on a subset of indoor videos, we measured the computation time by changing the distance of the quadrotor to understand the effect of the distance; and (ii) we analyzed the average running times needed to process indoor and outdoor frames, with respect to the number of stages and the thresholds.

**Figure 19:**
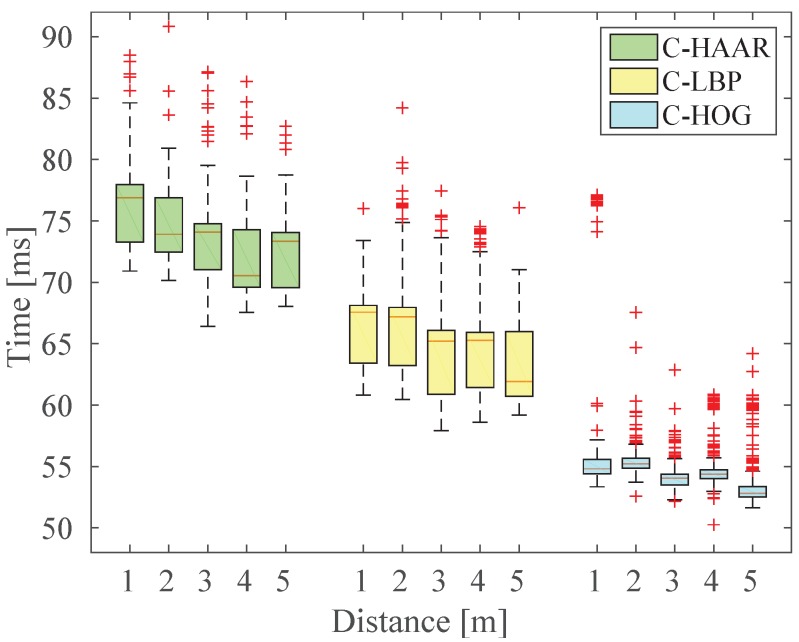
Change of computation time required to process one video frame with respect to the distance of the quadrotor.

For the first experiment, we have selected five videos from the yaw motion type for 1-, 2-, 3-, 4- and 5-m distances for the middle level height. In total, there were 1938 frames in these videos. We tested the performance of the classifiers having 19 stages at their default thresholds, as shown in Figure 19, with respect to the distance between the quadrotor and the camera. Although there are fluctuations, the time required to process a single frame shows an inverse correlation. This is so because as a quadrotor gets further away, its footprint in the image will decrease, and hence, the bigger scale detectors will reject the candidate windows faster, which will yield a speed up in the overall detection.

In our second experiment, we tested the running time performance of the classifiers with respect to the number of stages. This has been performed both for the classifiers at their default threshold, as well as with thresholds giving the maximum F-score (See Table 3 and Table 4).

**Figure 20:**
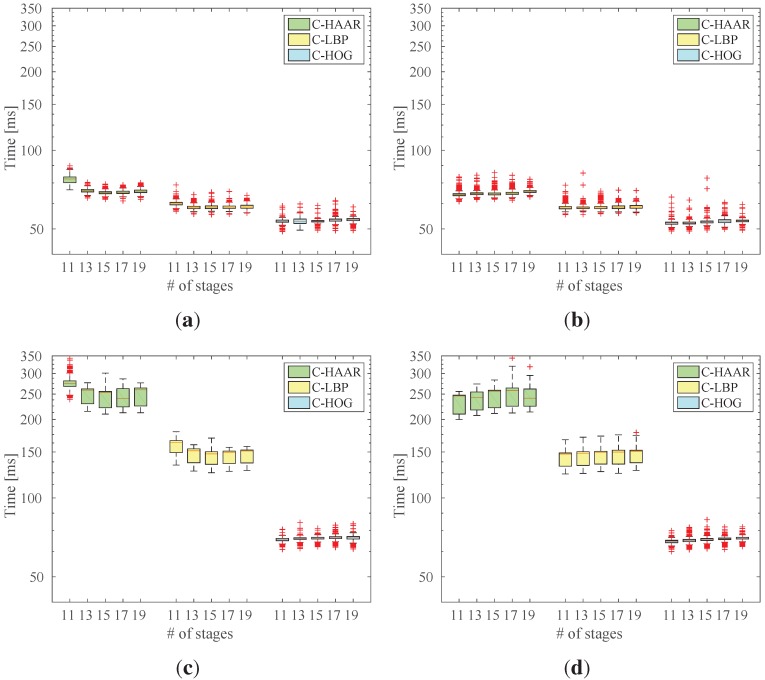
Analysis of time required to process one frame of (**a**,**b**) indoor and (**c**,**d**) outdoor videos. In (**a**,**c**), the classifiers are tested with their default thresholds, whereas in (**b**,**d**), the thresholds yielding the maximum F-score are used.

For indoor experiments, a subset of the indoor dataset consisting of videos from approach, down, lateral left-to-right and yaw-clockwise motion types containing 1366 frames in total was used. For the outdoor experiments, a total of 1500 frames from all motion types, namely calm, agile and moving background, were used. Figure 20 displays the resulting time performance distributions.

When we compare indoor and outdoor results, we observe that all three methods require more time to process outdoor frames. This increase reaches up to three times for C-HAAR and C-LBP. Outdoor frames are bigger than indoor frames by a factor of 1.15. This accounts partially for the increase in the processing time. However, the main reason is the higher complexity of outdoor background patterns, which manage to pass the early simple processing stages of the cascades more; thus, they consume more time before being identified as background.

When the results at the default thresholds and the maximum F-score thresholds are compared, we observe an increase in the time spent on the lower stages of C-HAAR and C-LBP. This is due to the increasing number of candidate bounding boxes that are later merged into the resulting bounding boxes. Both detection and merging of these high number of candidate bounding boxes causes the processing time to increase.

For the maximum F-score thresholds, processing time increases with the number of stages. This is an inherent result due to the increase in the number of stages.

**Figure 21:**
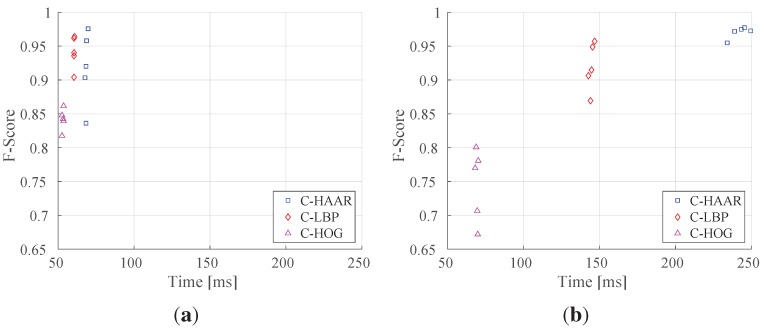
(**a**) Indoor and (**b**) outdoor scatter plots for F-score and mean running times. Each F-score value corresponds to a different classifier with different numbers of stages at the threshold resulting in the maximum F-score.

The scatter plots in Figure 21 display the distribution of F-scores with respect to the mean running times both for indoors and outdoors. The classifiers used in these plots are the ones giving maximum F-scores. The F-score values for C-HAAR and C-LBP are close to each other and higher than C-HOG. For C-HAAR, the F-score values are spread over a larger range for indoors, while the deviations in its mean time requirement increase for outdoors. Distributions observed for C-LBP for indoors and outdoors are similar to each other. The F-score values of C-HOG decrease and disperse over a wide range for outdoors, but the spread of its mean time requirement is very similar for indoors and outdoors.

### 5.7. Sample Visual Results

**Figure 22:**
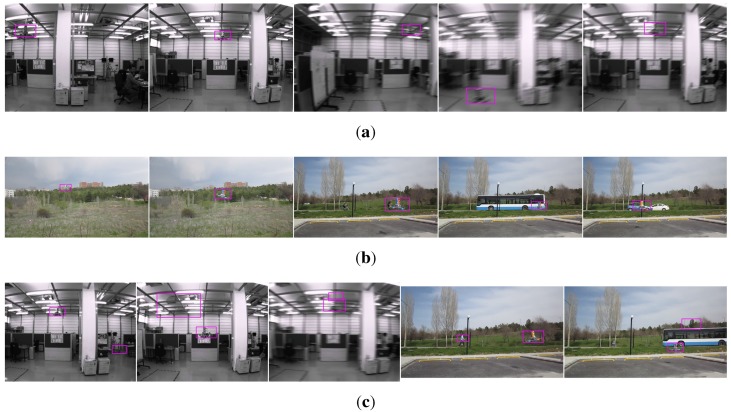
Successful detection and failure examples from indoor and outdoor experiments obtained using the best performing classifiers of C-LBP (only C-LBP results are provided for the sake of space). (**a**) Successful detections from indoor experiments.; (**b**) successful detections from outdoor experiments.; (**c**) failures from indoor and outdoor experiments.

In Figure 22, we present samples of successful detection and failure cases. These images are obtained using only the best performing C-LBP classifiers for the sake of space. C-LBP is remarkable among the three methods, since its detection and distance estimation performance is very high and close to that of C-HAAR. Furthermore, it is computationally more efficient than C-HAAR, both in training and testing. Three supplementary videos are also available (at http://www.kovan.ceng.metu.edu.tr/˜fatih/sensors/) as addendum showing the detection performance of C-LBP on video sequences from the indoor and outdoor test datasets.

The images in Figure 22a display the performance of the detector in an indoor environment that has extensive T junctions and horizontal patterns. The performance of the detector under motion blur is also displayed. Outdoor images in Figure 22b exemplify the outdoor performance of the detector where there are very complex textures, including also moving background patterns (pedestrians and various types of vehicles). When we look at the failures in Figure 22c, we observe that the regions including T junctions, horizontal patterns and silhouettes very similar to the quadrotor’s are the confusing areas for the algorithms.

## 6. Conclusions

In this article, we have studied whether an mUAV can be detected and its distance can be estimated with a camera through cascaded classifiers using different feature types. In order to demonstrate this in a systematic manner, we performed several experiments indoors and outdoors. For indoor evaluations, a motion platform was built to analyze the performance of the methods in controlled motions, namely in lateral, up-down, rotational and approach-leave motions. For outdoor evaluations, on the other hand, the methods were evaluated for cases where the mUAV was flown in a calm manner, an agile manner or with other moving objects in the background. The maximum detection distances of the methods are also analyzed with an outdoor experiment.

We evaluated the performance of three methods, namely C-HAAR, C-LBP and C-HOG, where, in each method, a different feature extraction approach is combined with the boosted cascaded classifiers and with a distance estimator utilizing SVR. Our experiments showed that near real-time detection and accurate distance estimation of mUAVs are possible. C-LBP becomes prominent among the three methods due to its: (1) high performance in detection and distance and time to collusion estimation; (2) moderate computation time; (3) reasonable training time; and (4) more robustness to the motion blur. When it comes to distance estimation, C-HAAR performs better, since it positions the bounding boxes more accurately compared to the other methods. On the other hand, our time analysis reveals that C-HOG is the fastest, both in training and testing.

We have demonstrated that an mUAV can be detected in about 60 ms indoors and 150 ms outdoors in images with 1032×778 and 1280×720 resolutions, respectively, with a detection rate of 0.96 for the F-score, both indoors and outdoors. Although this cannot be considered real time, a real-time performance with cascaded classifiers is reachable, especially considering that the implementations are not optimized. We also showed that distance estimation of mUAVs is possible using simple geometric cues and the SVR; even the change in the pose of the quadrotor or the camera results in different bounding boxes for the same distance between mUAV and the camera.

The performance of detection can be improved significantly when combined with tracking, e.g., by employing tracking-by-detection methods [89,90,91]. Such methods limit the search space of the detector in the next frame(s) by using the properties of the current and previous detections. This can improve both the running time and the detection performance substantially.

Cascaded approaches are known to generalize rather well with the increase of the number of objects. By looking at simple, fast, yet effective features at multiple stages to minimize false positives and to maximize detection rates, successful applications on complex and challenging datasets with many exemplars of the same class have been reported [36,37,92]. These indicate that, for mUAV detection, cascaded approaches are very suitable, even if many mUAV variants with appearance characteristics are included.

## Supplementary Files

Supplementary File 1
